# Successful avatrombopag combined with cyclosporine treatment for carboplatin/pegylated liposomal doxorubicin/bevacizumab-induced acquired amegakaryocytic thrombocytopenia in a patient with recurrent ovarian cancer: case report

**DOI:** 10.3389/fonc.2024.1253230

**Published:** 2024-02-09

**Authors:** Weikang Meng, Jinsheng Hua, Jiabing Wang

**Affiliations:** ^1^ Department of Hematologic Oncology, Taizhou Municipal Hospital, Taizhou, China; ^2^ Department of Pharmacy, Taizhou Municipal Hospital, Taizhou, China

**Keywords:** acquired amegakaryocytic thrombocytopenia, carboplatin/pegylated liposomal doxorubicin/bevacizumab, avatrombopag, cyclosporine, recurrent ovarian cancer

## Abstract

Carboplatin/pegylated liposomal doxorubicin/bevacizumab is an accepted standard anti-cancer treatment option for recurrent ovarian cancer. However, the occurrence of adverse events associated with this therapeutic regimen limits its continued clinical use. Among these adverse events, acquired amegakaryocytic thrombocytopenia is a rare but often potentially life-threatening adverse effect, and is intolerant to multiple treatment approaches. We report, for the first time, the successful treatment using avatrombopag combined with cyclosporine in one case of carboplatin/pegylated liposomal doxorubicin/bevacizumab-induced acquired amegakaryocytic thrombocytopenia, which was refractory or intolerant to glucocorticoids, intravenous immunoglobulin, recombinant human thrombopoietin, androgen, and even thrombopoietin receptor receptor agonist eltrombopag and herombopag. To date, this case manifests as normal platelet counts that are independent of transfusion. Our findings suggest that this combination is a potential and valuable alternative in acquired amegakaryocytic thrombocytopenia.

## Introduction

Ovarian cancer is one of the common gynecological reproductive system tumors in women globally, and its mortality rate ranks first among all kinds of gynecological tumors (1). Cytoreductive surgery and platinum/taxane doublet chemotherapy are the main regimen for newly diagnosed ovarian cancer; however, 70%–80% of patients with advanced cancer relapse, even if they achieve complete remission after management. At relapse, carboplatin/pegylated liposomal doxorubicin (PLD)/bevacizumab is a new standard treatment option for recurrent ovarian cancer (1). In this phase 3 clinical trial of recurrent ovarian cancer, carboplatin/PLD/bevacizumab-induced adverse events frequently occur and can potentially affect all organs. The most common grade 3/4 adverse events were hypertension (28%) and neutropenia (12%), while acquired amegakaryocytic thrombocytopenia (AAT) is rare (1). In this study, we report, for the first time, the successful treatment using avatrombopag combined with cyclosporine in one recurrent ovarian cancer patient with carboplatin/PLD/bevacizumab induced AAT.

## Case report

A 44-year-old-female was diagnosed with ovarian serous carcinoma stage IV in August 2020. After hysterectomy and oophorectomy and six cycles of combined chemotherapy (platinum and taxane), her conditions were under control. Unfortunately, she was confirmed to have a recurrence of ovarian malignancy on 8 March 2022, and she was administered six cycles of carboplatin/PLD/bevacizumab. After the fifth chemotherapy cycle, her condition was under control, but she had nose bleeding and was admitted to the emergency room on 1 August 2022 (day 1). Admission laboratory findings revealed a platelet (PLT) count of 20×10^9^/L (normal: 125–350×10^9^/L), a white blood cell count (WBC) of 2.3×10^9^/L (normal: 3.5–9.5×10^9^/L), and a hemoglobin (Hb) level of 76 g/L (normal: 110–150 g/L). She was diagnosed with myelosuppression and given recombinant human thrombopoietin (rh-TPO, 15,000 U/day), human granulocyte colony stimulating factor (HGCSF, 5 μg/kg/day), and platelet transfusion (PTT). The levels of PLT, WBC, and Hb started rising and she was discharged after 20 days. On 8 September 2022 (day 39), she finished the last cycle of a reduced dose combined chemotherapy (details of the magnitude of the dose reduction were not available) because of myelosuppression. Unfortunately, she developed thrombocytopenia (PLT 41×10^9^/L) and anemia (Hb 76 g/L) and was hospitalized on 17 September 2022 (day 48). rh-TPO, PTT, and red cells transfusion (RCT) were administered, and her PLT remained at 27~49×10^9^/L during the hospitalization period. On 30 September 2022 (day 61), she was discharged and her PLT remained at 70~117×10^9^/L.

Two months later, repeated routine blood examination showed a decreased PLT of 35×10^9^/L. She was hospitalized again on 27 November 2022 (day 117) and treated with rh-TPO daily for 7 consecutive days without good response; her PLT remained at 35×10^9^/L. Bone marrow morphology and flow cytometry showed an absence of megakaryocytes, with no significant abnormal presentation of other cell linages; flow cytometry of bone marrow and karyotype did not show any abnormalities. Therefore, AAT was considered. Testosterone undecanoate (TU, 120 mg/day) and eltrombopag (ELT, 50 mg/day) were added on 4 December 2022 (day 128), followed by PTT and HGCSF. PLT gradually rose to 59×10^9^/L. She had no bleeding gums; however, no response was observed, and the PLT continuously decreased to 20×10^9^/L. She asked to be discharged on 7 January 2023 (day 158).

**Figure 1:**
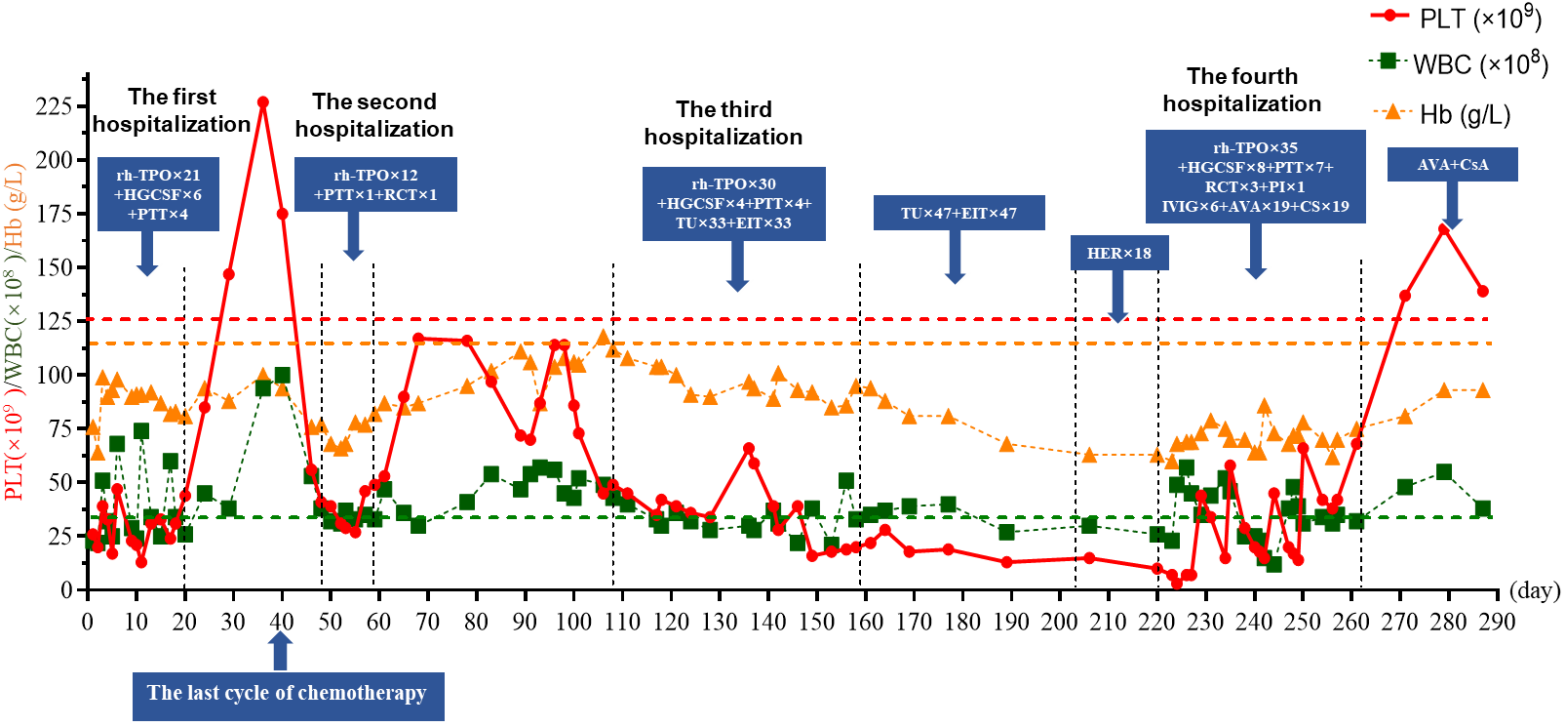
Changes in PLT, WBC, and Hb over the course of treatment.

After discharge, the patient received oral TU and ELT for treatment, which lasted for 47 days, but they showed limited responses. The patient was transitioned to oral herombopag (HER, 7.5 mg/day); however, this also showed no curative effect. On 13 March 2023 (day 223), the patient was readmitted for recurrent severe thrombocytopenia with bleeding gums. Laboratory experiments revealed a PLT level of 7×10^9^/L. After admission, she received rh-TPO, HGCSF, PTT, and RCT, without a good response; PLT decreased to 3×10^9^/L on 14 March 2023 (day 224). She began to receive intravenous immunoglobulin (IVIG, 20 g/day) with dexamethasone (10 mg/day), tranexamic acid 0.8 g daily, rh-TPO for 5 days, and one plasma infusion. Her PLT increased to 44×10^9^/L and the bleeding gums improved. Her PLT did not improve further and was between 15×10^9^/L and 58×10^9^/L, despite the re-administration of IVIG, PTT, and rh-TPO. On 1 April 2023 (day 242), avatrombopag (AVA) 60 mg daily and cyclosporine (CsA, 20 mg/day) were added. Three days later, laboratory experiments revealed a PLT level of 45×10^9^/L. However, PLT deteriorated again for 3 consecutive days and she was treated with IVIG and PTT; meanwhile, Rh-TPO, AVA, and CsA were given daily. Surprisingly, her PLT started to improve and remained at 38~66×10^9^/L. On 21 April 2023 (day 261), the PLT increased to 69×10^9^/L; she was discharged and received oral AVA and CsA for treatment and maintenance. The CsA daily dose was adjusted with a trough concentration maintained at 100–200 ng/mL. Fortunately, until the time of writing this case report, the patient’s condition is well controlled. Blood routine examinations showed that the PLT increased to normal levels and was maintained (**Figure 1**). Moreover, there were no adverse events observed with any of the thrombocytopenia treatments.

## Discussion

AAT is an extremely rare hematological disorder characterized by the complete absence of megakaryocytes in the bone marrow, accompanied by severe thrombocytopenia, and distinct from the dysfunction of megakaryocyte maturation in immune thrombocytopenia (ITP) (2, 3). Clinically, patients with AAT are often initially misdiagnosed as having ITP but do not usually respond to ITP first-line therapy (i.e., corticosteroids (CS) and IVIg) (2). AAT usually presents with bleeding, bruising, and severe thrombocytopenia; however, because of the significantly increased risk of bleeding from vital organs, AAT is often severe and life-threatening (2, 3). So far, the pathophysiological mechanism of AAT remains unclear. However, two theories are widely accepted, including patients with autoimmune diseases and intrinsic defects of MK progenitor cells caused by inhibition of megakaryocyte colony-forming units (MK-CFU) in serum AAT patients *in vitro* or by action of cytotoxic T lymphocytes in AAT patients on MK progenitor cells (2, 3).

Although there is currently no standard treatment, Hoffman et al. describe an empirical approach to AAT based on the mechanism of thrombocytopenia (4). CS, IVIG, cyclophosphamide, and CsA were found to be effective in AAT patients with presumed or measured antibodies against MK-CFU (2, 3), while patients with T-cell-mediated megakaryopoiesis inhibition responded well to anti-thymocyte globulin (ATG) (2, 3). However, the fact remains that most ATT patients tend to be intolerant to empiric treatments. Published reports show that most ATT patients treated with CsA show a sustained and long-lasting response relative to CS therapy (2). Meanwhile, newly FDA-approved oral thrombopoietin receptor agonists (TPO-RAs) for the treatment of immune ITP, such as ELT, HER, and AVA, are increasingly being recognized as standard treatment for AAT patients (2). More importantly, Roeser et al. found that that monotherapy or combination therapy with TPO-RAs or CsA is a potential treatment option for refractory thrombocytopenia and ATT (2). Furthermore, we reviewed case reports published in the Pubmed database that demonstrate successful treatment of AAT with oral TPO-RAs and (or) CsA alone or in combination (**Table 1**) (4–13), suggesting that this strategy can be a valuable alternative in AAT.

**Table 1:** Successful treatment of AAT with oral TPO-RAs and (or) CsA alone or combination published in the Pubmed database between the years 2000–2022.

Study	Age (yrs)/gender	Primary disease	Inducers	Initial presentation	Initial platelet count (×10^3^/μL)	Initial therapy	Definitive therapy	Platelet count at follow-up (×10^3^/μL)	Follow-up duration (mo)
Agarwal et al., 2006 (4)	48/F	–	–	Easy bruising	25	Steroids, IVIG, recombinant IL-11	CsA 250 mg BID	225	4
Cela et al., 2010 (5)	55/M	Systemic lupus erythematosus	–	Chest pain, thrombocytopenia	28	Steroids, rituximab, CsA	Eltrombopag 50 mg/d	179	1.5
Jain et al., 2012 (6)	8/M	–	–	Petechiae, hematuria	20	Steroids	CsA 12 mg/kg/d	160	9
Hashimoto et al., 2016 (7)	61/F	Rheumatoid arthritis、type 2 diabetes	–	Intra-oral hematomas, ecchymosis, petechiae, thrombocytopenia	4	–	CsA 150 mg BID	100	12
Onuki et al., 2016 (8)	67/F	Invasive thymoma	–		6	–	CsA 300 mg/d	204	7.5
Iyama et al., 2020 (9)	54/F	Advanced pancreatic cancer	Nivolumab	Thrombocytopenia	12	Steroids, IVIG	CsA 3 mg/kg	125	1
Roy et al., 2020 (10)	50/M	Chronic idiopathic thrombocytopenic purpura	–	Easy bruising, thrombocytopenia, prolonged bleeding after trauma	19	Steroids	CsA 150 mg BID (2.5 mg/kg/d) to 200 mg BID	63	10
Suyama et al., 2021 (11)	78/M	Squamous cell carcinoma	Durvalumab	Thrombocytopenia	7	Steroids	Eltrombopag 25 mg to 50 mg	223–480	9
Tian et al., 2021 (12)	15/M	–	–	Thrombocytopenia	1	Rh-TPO, steroids, IVIG	Eltrombopag 50 mg/d and CsA 3 mg/kg/d	>150	8
Tu et al., 2022 (13)	67/M	Ureter neoplasm with right hydronephrosis and retroperitoneal lymph node metastasis	Tislelizumab	Thrombocytopenia and petechiae	48	Rh-TPO, steroids, IVIG, CsA, eltrombopag	Avatrombopag 40 mg daily	100	2
	71/F	Bladder cancer	Tislelizumab	Thrombocytopenia and petechiae	26	Rh-TPO, steroids, IVIG, eltrombopag, G-CSF	Avatrombopag 40 mg daily	94	2

“–” means none.

Until now, there have been few studies on the induction of AAT by chemotherapy and targeted therapy, and the corresponding treatment methods are still unclear. In our case, we, for the first time, reported that a patient with carboplatin/PLD/bevacizumab-induced AAT, refractory to CS, IVIG, rh-TPO, androgen, and even ELT and HER, responded to AVA and CsA. AVA is an oral, small molecule TPO-RA that stimulates the proliferation and differentiation of megakaryocytes in bone marrow progenitor cells, thereby increasing PT production. It does not compete with TPO to bind TPO receptors and has an additive effect with TPO on PLT production (14). Unlike PTT, AVA is predictable in increasing PLT and can be used as an alternative treatment for PTT. CsA can inhibit the activity of cytotoxic T lymphocytes and helper T cells, and then promote the hematopoietic function of bone marrow. During AVA and CsA treatment, the patient achieved remission without obvious side effects, suggesting that this combination is a potential and valuable alternative in AAT, although pooled data indicated that some patients experienced headache, fatigue, peripheral edema, fever, fatigue, abdominal pain, nausea, and elevated creatinine and urea, etc.

Up to now, no standard therapy for ATT has been consensually defined to date. However, Anais et al. demonstrated that CsA was very effective and could be considered as a first-line therapy. Although having limited experience in this rare setting, TPO-RAs may also be effective and should be considered in refractory cases, in monotherapy, or in association with CsA (2). Moreover, the cases of successful treatment of AAT with oral TPO-RAs and (or) CsA alone or in combination that we included, as well as the case in this study, illustrate that TPO-RAs alone or in combination with CsA could be an effective alternative therapy for ATT (4–13), although the effectiveness and safety of this treatment regimen requires more research, such as clinical trials. Meanwhile, this alternative therapy does not currently meet international/local consensus criteria; we thus encourage more studies on the topic.

In conclusion, carboplatin/PLD/bevacizumab-induced AAT is rare but often serious and is refractory to multiple treatments. This study presents AVA and CsA as a potential, safe treatment and good approach for patients with AAT who have failed recommended or empirical treatment. After failure of AVA combined with CsA, the addition of antithymocyte globulin or allogenic stem cell transplantation may be effective for the patient (15, 16).

## Data availability statement

The raw data supporting the conclusions of this article will be made available by the authors, without undue reservation.

## Ethics statement

The studies involving human participants were reviewed and approved by the Clinical Ethics Committee of Taizhou Municipal Hospital (LWYJ202300125). The patients/participants provided their written informed consent to participate in this study. Written informed consent was obtained from the individual(s) for the publication of any potentially identifiable images or data included in this article.

## Author contributions

JW designed the study. JH and WM collected the data and performed the data analyses. JW wrote the original manuscript. JW revised and supervised the paper. All authors contributed to the article and approved the submitted version.
